# Care trajectories of people with mood disorders in Quebec using latent class and latent profile analysis methods

**DOI:** 10.1016/j.xjmad.2024.100101

**Published:** 2024-12-14

**Authors:** Christian Roger Claver Kouakou, Matea Bélan, Thomas G. Poder, Maude Laberge

**Affiliations:** a Université Laval, Canada; b Université de Sherbrooke, Canada; c École de santé publique, Université de Montréal, Canada; d CR-IUSMM, Centre de recherche de l′Institut universitaire en santé mentale de Montréal, Canada; e Vitam, Centre de recherche en santé durable, Canada; f Centre de recherche du CHU de Québec, Canada

**Keywords:** Mood disorders, Care trajectories, Latent class analysis, Latent profile analysis

## Abstract

The prevalence of mood disorders has increased globally. People with mood disorders have been found to use more health services than the general population, although a mood disorder diagnosis does not necessarily entail utilization of health services. This heterogeneity in health services utilization could make it difficult for governments to plan resources to meet the needs of people with mood disorders. A patient-level linked database from residents of Quebec, Canada was used to model care trajectories of people who self-reported having been diagnosed with a mood disorder. The data from the Canadian Community Health Survey were linked to health administrative data for a 21-year period. We used latent class analysis and latent profile analysis to group people into categories. Four care trajectories were identified using the latent class analysis: 1) people who only used services of a general practitioner; 2) people having seen a psychiatrist or having at least one ED visit or hospitalization; 3) people consulting other types of specialists; 4) null utilization. The latent profile analysis on medical services yielded four profiles, with average numbers of services of 41, 33, 7, and 1, while that on hospitalization yielded two profiles, with 20 % of the population having had at least one hospitalization and the remainder none. By classifying people into service utilization groups, these methods enable determining needs for a given population and can support resource allocation for health care decision makers.

## 1 Introduction

Mood disorders (MD) can be defined as a “group of psychiatric illness that can simultaneously affect one’s emotions, energy and motivation” [1]. There are many types of MD, but three are most common: depression, bipolar disorder and dysthymia. A broad range of symptoms characterize these diseases including, but not limited to, a change in appetite, loss of weight, difficulties concentrating, suicidal behaviour, low self-esteem, insomnia or hypersomnia, and logorrhea. In Quebec, Canada’s second-most populous province, MD prevalence in the adult population increased from 5.5 % in 2015 to 6.2 % in 2020 [2]. The age groups with the highest prevalence are 18–34 years old and 50–64 years old, with women seemingly more affected than men.

MD can be caused by many factors, with trauma exposure, as well as emotional and sexual abuse, being the most commonly associated with depressive symptomatology [3]. Over the course of life, some medical conditions have shown to have a significant impact on mood. For example, studies have showed that during breast cancer and in the perinatal period, women were more likely to experience MD, and factors such as stress, low income and low education can exacerbate the severity of MD [4], [5].

Several studies examined health care utilization for people with MD. For instance, having a mood disorder has been associated with a higher utilization of emergency departments in the United States [6] and higher hospitalization costs [7]. In the United States, a country without universal health coverage, people with MD are less likely to have health insurance. Despite experiencing more barriers to care, they have frequent medical visits compared to the general population and people with other chronic conditions [8]. However, having MD does not mean that one has a high utilization of health services. A study conducted in Ontario, Canada, reported a null utilization of health services for about half of people with MD; in addition, living in a rural or urban setting was not a factor in health services utilization [9]. In another study using Canadian data, 60 % of respondents with MD reported using medical services [10].

MD can also co-occur with other chronic conditions and studies suggest that, in such cases, they are associated with a higher utilization of health services compared to people with the chronic condition but with no co-occurring MD. Such results were reported in an analysis of Canadian Communities Health Survey (CCHS) for people with obesity [11] and in a study using administrative data from Manitoba for people with multiple sclerosis [12].

The types of services used may be more heterogenous than that captured in health administrative databases, which are often limited to physician and hospital services. For instance, in a study analyzing data from the 2003 CCHS, among people with MD who sought help, half used “conventional mental health services” while 21 % used “natural health products specifically for emotional, mental health, and drug or alcohol use problems” [10].

The empirical data suggest that people with MD have a higher utilization of health services than people without MD. In a context where MD diagnoses are increasing [13], [14], it is important to identify patterns of utilization, which will enable a better planning of resources. Studies examining utilization of people with mental health or mood disorders are limited and heterogeneous. In a study of care trajectories of Chinese people with depressive symptoms over a 12-month period, seven classes were identified, including trajectories of remission, of stabilization, and of deterioration [15]. In Australia, a study on utilization of health services of women with mental health over a seven-year period found six classes of trajectories, of which one was considered a high users’ class that included 7.2 % of the study population [16]. Examining health services utilization data of people prior to their schizophrenia, Vanasse et al. [17] identified six trajectories, with one trajectory shared by most of the study population and which consisted of mainly young men with no to low use of services. Four trajectories were identified in people with bipolar depression, with the largest proportion of the study population in a class of low utilization [18]. A systematic review on mental health service seeking among young people suggest that there is not one single trajectory, but instead complex trajectories, with diverse contacts [19]. Most studies examining utilization are cross-sectional and only examine a limited period, such as one year. MD are chronic conditions, but utilization of services may vary over time.

The care trajectory is a concept that emerged in the past few years and has been increasingly used to examine the utilization of health services in given populations [20]. Although there is not a single accepted definition of the concept, it generally involves examining resource utilization over a pre-defined period (cross-sectionally or longitudinally), whether it be in terms of the types of services used, the order in which they are used, the delays between their use, or the intensity of the utilization. Although we consider “care trajectory” as a phrase that does not properly illustrate the concept, which is operationalized with health services utilization rather than care, we will nevertheless use this term henceforth, to be consistent with the literature. We hypothesize from our review of the literature summarized above on the use of health services by people with MD that there is heterogeneity in health services utilization, and hence not a single care trajectory, but various care trajectories. Grouping people in care trajectories could help in predicting resources needs and hence improve planning. Recent years have seen the development of classification methods for care trajectories, which are described by Nguena Nguefack et al. [21]. Briefly, the authors classified trajectory modelling methods into three groups: parametric (growth mixture modelling), nonparametric (cluster analysis and sequence analysis), and semi-parametric (group-based trajectory modelling [GBTM], latent class analysis, and latent transition analysis) approaches. Latent transition analysis is generally used with longitudinal data to examine transition after an event, such as care trajectories after COVID-19 [22], while latent class analysis can be used with cross-sectional data. Latent class analysis has been also used to group people with MD by symptoms [23], [24].

The objective of this study was to apply two trajectory modelling approaches, i.e., latent class analysis (LCA) and latent profile analysis (LPA) to identify care trajectories for people with MD. LCA and LPA are methods in which we consider characteristics of a population that are a priori unobserved. Since we do not know how people with MD behave in term of health services utilization, using these methods was deemed appropriate.

## 2 Methods

### 2.1 Data sources

We used longitudinal data from The Care Trajectories–Enriched Data Cohort (TorSaDE) [25], a unique database created through linking of data from various databases. First, it consists of respondents to at least one of the four cycles of the Canadian Community Health Survey (CCHS), i.e., 2007–2008, 2009–2020, 2011–2012, and 2013–2014, who reported being residents of Quebec, Canada, and who agreed to have their data collected in the CCHS shared with the Ministry of Health and Social Services, the Institut de la statistique du Québec (ISQ), Health Canada and the Public Health Agency of Canada for research purposes. It should be noted that more than 92 % of participants agree to such sharing and that these organizations have strict policies to ensure that participants are not identified [25]. The self-reported data from the CCHS participants were linked to Quebec health administrative databases from the Quebec public health insurance, the Régie de l’assurance maladie du Québec (henceforth, RAMQ) for a period covering 21 years (1996–2016). Hence, TorSaDE is a cohort that includes both self-reported data with a wide array of questions and 21 years of data for each individual on their utilization of health care services, including medical services and services provided in hospital settings, for all participants. For those insured by the Quebec public medication insurance, Régime public d’assurance médicament (RPAM), it also includes prescription drugs.

The RAMQ is a universal health insurance that covers every resident of Quebec with a few exceptions, such as newly arrived residents (less than three months), people living on Indigenous reserves or refugees, who are covered by other insurances plans. These data allow to match in a unique file, self-reported health and socioeconomic data with observed health services utilization data.

### 2.2 Study population

A subset of TorSaDE participants was used, i.e., adults with MD. We considered two options to identify those participants. One option was to use a diagnostic from a third party, i.e., a physician, reported in medical visits and available in the RAMQ data. The second option was to use the participants’ answers to the questions in the CCHS asking respondents whether they had been diagnosed by a health professional with a MD, such as depression, bipolar disorder, mania, or dysthymia. Inconsistencies between those two methods using data from TorSaDE have been reported elsewhere for other conditions [26]. We decided to use self-reporting for multiple reasons. First, it corresponds to the situation lived by the respondent at the time of completing the CCHS and, as such, enables using other variables collected in the CCHS. Second, experts we consulted considered that diagnoses available in the RAMQ databases may be of poor quality in the case of MD. One of the reasons for this was that each medical visit has only one diagnosis, even though people may consult for more than one issue. Hence, we could be underestimating the number of participants with MD. Third, people with MD may not use medical or hospital services in the period selected to identify them, which would lead to under-representation of low service users. Finally, people may have had a short-lived depression recorded in the RAMQ data but have recovered at the time of completing the CCHS, hence including those may cause overestimation. Other researchers made the same choice for individuals with anxiety disorders [27].

### 2.3 Study design

We conducted LCA and LPA for people with MD. For each person, we considered their positive answer to the question about having been diagnosed with a MD as their index date (i.e., when they completed the CCHS). For LCA, the follow-up period consisted of the five years prior to the reported diagnosis and the two years following this date. For instance, if a person reported a MD in October 2009, the follow-up period was from October 2004 to October 2011. The choice of the follow-up period was based on having RAMQ data up until 2016, which was two years after the last CCHS, and the desire to have a long period over which to examine services utilization, hence five years before the index date. We chose to examine monthly services utilization, which means we had a total follow-up period of 85 months, i.e., seven years, plus the month of the reported diagnosis. A daily or weekly frequency would certainly have represented too many data points where individuals would have had no contact with the health system. For LPA, we examined yearly utilization and included the year of the reported diagnosis, which means the follow-up period was eight years (five years before diagnosis, year of diagnosis, two years after). Both LCA and LPA are statistical techniques that consist of segmenting a given population based on observable data. The fundamental difference between these two techniques lies in the nature of the variables (discrete or continuous).

### 2.4 Variables

For LCA, outcome variables consisted of measures of different types of health services used and all health services were considered in our analyses, regardless of whether they were related to the MD. Our outcome variables were classified into five categories: consultation with a family physician (general practitioner), consultation with a psychiatrist, consultation with another specialist (other than a psychiatrist), hospitalization and ED visits, and null utilization. A null utilization was considered since it represents a category of health services utilization. Since hospitalizations were infrequent, we decided to combine hospitalizations and ED visits.

For LPA, we used two outcome variables: the number of medical services, and the number of hospitalizations.

All outcome variables are not specific to mood disorders. We decided not to limit the utilization to services for mood disorders based on discussion with experts. They expressed that when a patient presents at the emergency, the reason for the visit may be a symptom of the MD, but the MD may not be recorded as the reason for the visit. In addition, clinicians and patients alike mention that the MD may affect their capacity to manage other health conditions, and hence some utilization of services would be indirectly related to MD. They considered that it was important to include all utilization of services and not only services that were recorded as directly for the MD. As mentioned earlier, diagnosis data is also of poor quality in physician billing data, which is used to identify medical consultations.

We used covariates in the regressions. Most of these variables were individuals’ characteristics and came from the CCHS: demographic and socioeconomic variables (e.g., age, highest level of education attained, gender, income, marital status, household size).

### 2.5 Statistical analyses

As stated earlier, trajectories for people with MD were analyzed using two methods: LCA and LPA. These “are techniques that aim to recover hidden groups from observed data” [28]. LCA and LPA are relatively recent in health services research, but their use is increasing to identify categories within populations of health services users. For instance, LCA has been used for people with tuberculosis [29] or alcohol disorders [30], and included with TorSaDE data for geriatric emergency department users [31]. Others used LPA to study services utilization in people with substance use disorders [32], in harm-reduction service utilization [33] or dental care [34], to name a few. LCA and LPA are members of the Gaussian finite mixture model family. The difference is that LPA uses continuous variables, while LCA uses binary variables. We decided to use latent class and latent profile methods over sequential analysis, since they allow us to explore the underlying structure of our data and understand how individuals can be grouped together based on similarities in their responses. Sequential analysis, on the other hand, is typically used to analyze time-ordered data and detect patterns or changes over time within individual units.

In the LCA, all five outcome variables, namely consultation with a family physician, consultation with a psychiatrist, consultation with another specialist (other than a psychiatrist), hospitalization and ED visits, and null utilization, were transformed into binary variables. If the service was used, then the variable took the value 1. If it was not used, then the value 0 was applied. Utilisation of medical and hospital services were grouped by calendar month. We did not consider how many times a participant had used a particular service within a month. All we looked for was whether the service was used at least once or not. Hence, for LCA, we constituted a database in which we created binary occurrence variables for each outcome and period (i.e., for each month), which means that for the 85 months of the study period we had a total of 375,785 individuals-months.

In the LPA, for the same five outcome variables (used in the LCA), we determined the number of times of occurrence each month. In case of a null utilization in a given month, the variable took a value of 0. Considering the longitudinal nature of the data, we used a growth mixture model (GMM) to predict groups among respondents and consider potential intra-group heterogeneity. Each hospitalization was identified with a unique identifier, which we used to regroup the hospitalization episode of the person, including diagnosis, medical services, intensive care, and interventions. Averages were calculated for each person and for each year before, during and after the index date. We merged the hospitalization database with the CCHS database to create databases with the information required for the estimations, which meant creating of five databases for each element related to what happened during the hospitalization: diagnoses, medical services, interventions and intensive care (if applicable). Hence, for 4421 individuals and eight years, we had a total of 35,368 individuals-years.

For LCA, we tested various models while varying the number of classes between two and five each time. Statistical criteria were used for the selection of the most appropriate model and the number of categories. We used the Bayesian information criterion (BIC) and the Akaike information criterion (AIC) [35] to select the most appropriate model and the number of classes.

To determine the profiles, we estimated a finite mixture model, which required specifying the number of profiles and the form of the distribution function. We used a negative binomial function for the number of services and diagnoses. A Poisson distribution was also considered, but it was not suitable to the distribution of our variables, and specifically to the overdispersion that we observed. Our approach was similar to the one with LCA, i.e., we tested various models with varying the number of profiles and used the AIC and the BIC to decide on the most appropriate.

The marginal mean and probability of each class and profile were calculated. Classes and profiles were graphed to highlight groups of care trajectories among participants.

Analyses were conducted using RStudio for the LCA (with the poLCA package) and Stata© for the APL. Since PoLCA does not support 0 values, we transformed these into 2.

## 3 Results

### 3.1 Participant characteristics

Table 1 presents the characteristics of the study population which consisted of the 4421 Quebec adults who reported having a MD in the CCHS between 2007 and 2014. The average age was 47 years old and women represented a large majority (61 %) of the study population. For the most part, respondents lived in a two-people household, and the average annual income was close to 50,000 Canadian dollars (CAD). Overall, more than half of the households earned more than 40,000 CAD. A third of respondents were single or never married, whereas 45 % were married or common-law partners. One out of five people were widowed, separated or divorced. More than 80 % of respondents had no child aged under 12. More than half had a post-secondary diploma or went to university; nearly a quarter had secondary school or less as their higher education level. Almost all respondents were born in Canada or in a country from the Americas. When self-assessing their health, two thirds of respondents reported good to excellent physical and mental health, and a third reported fair or bad health. Regarding stress, 47–50 % thought life or work was fairly or extremely stressful. Overall, the average health state measured with the health utility index HUI3 was at 0.682, which is lower than the 0.863 score reported for the Canadian population [36]. Finally, for our outcome variables, 58 % of the respondents did not used any health services in the study period, and only 5 % saw a psychiatrist.

**Table 1:** Descriptive statistics of the population with mood disorders in TorSaDE.

**Socioeconomic characteristics**	**Mean (sd)**
	(N = 4421)
*Gender*	
- Female	61.48 %
- Male	38.52 %
*Age (years)*	46.81 (16.24)
*Household size*	2.36 (1.31)
*Household income (CA$)*	48,684 (28,766)
< 40,000 CAD	44.61 %
≥ 40,000 CAD	55.39 %
*Marital status*	
- Married or common-law partner	45.40 %
- Widowed, separated, divorced	20.70 %
- Single, never married	33.90 %
*Child* < *12 yo*	0.27 (0.67)
- No child	82.67 %
- At least 1 child	17.33 %
*Education*	
- High school or less	23.10 %
- High school diploma with post-sec.	13.83 %
- Partial post-secondary studies	8.23 %
- Post-secondary diploma or university	54.83 %
*Birthplace*	
- Canada & America	90.97 %
- Rest of the world	9.03 %
**Self-assessment of health**	
*Overall health*	
- Very good to excellent	28.39 %
- Good	37.51 %
- Fair to bad	34.10 %
*Mental health*	
- Very good to excellent	25.64 %
- Good	40.97 %
- Fair to bad	33.39 %
*Perceived stress in life*	
- Not very to not at all stressful	18.42 %
- A little stressful	34.28 %
- Fairly to extremely stressful	47.30 %
*Stress at work*	
- Not very to not at all stressful	17.91 %
- A little stressful	31.92 %
- Fairly to extremely stressful	50.18 %
*Health utility index (HUI3)*	0.6819 (0.2945)
**Monthly health services utilization**	
- No service	58.10 %
- General practitioner	16.46 %
- Psychiatrist	5.30 %
- Other specialist	14.06 %
- Hospitalization & emergency	6.08 %
*Number of services used*	
0	58.10 %
≥ 1	41.90 %

Weighted mean with Statistics Canada weight

### 3.2 Latent class analysis

We reported the results of the statistical tests on models using three to five classes in Table 2 below.

**Table 2:** Post-estimation statistical optimization criteria.

	3 classes	4 classes	5 classes
AIC	1096,158	971,747.3	971,759.3
BIC	1096,342	971,996.5	972,073.5
G^2^	183,506.9	59,084.44	59,084.44
X^2^	157,468	42,762	42,762

AIC: Akaike information criterion, BIC: Bayesian information criterion, G^2^: G-square for goodness of fit, X^2^: Chi-square.

Based on the results of the post-estimation, we selected the model with four classes for the LCA.

We reported utilization characteristics of classes in Table 3. Class 1 (n = 760) corresponds to people who had services with general practitioners. Class 2 (n = 762) corresponds to people who had a consultation with a psychiatrist and to people who were hospitalized/had ED visits. In class 3 (n = 238), all people consulted other specialists, and finally class 4 (n = 2661), with more than half of the respondents, corresponds to people who did not use any medical services. The LCA allowed to distinguish two sub-groups within the category of miscellaneous consumers (general practitioner and other specialists).

**Table 3:** Characteristics of utilization in each class.

	Class 1	Class 2	Class 3	Class 4
Null utilization	0	0	0	100 %
General practitioner	100 %	0	0	0
Psychiatrist	0	46.6 %	0	0
Other specialist	0	0	100 %	0
Hospitalization or ED visit	0	53.4 %	0	0

The comparison of the descriptive data between the four classes in Table 4 shows that classes 1 and 2 have more women (70.39 and 71 % vs. 56.30 and 61.03 %) and older people (56.98 and 52.22 % vs. 48.11 and 48.11 and 48.89 %) than the two other classes (i.e., classes 3 and 4). Class 3 members have a lower average income (33,277 CA$ vs. > 40,000 CA$) and are more likely to be single or never married (40.34 % vs. < 35 %). Members of classes 3 and 4 were more educated.

**Table 4:** Descriptive data comparison between classes after LCA analysis.

	**Class 1 (N = 760, 17.2 %)**	**Class 2 (N = 762; 17.2 %)**	**Class 3 (N = 238, 5.4 %)**	**Class 4 (N = 2661, 60.2 %)**	**p-value**
**Socioeconomic characteristics**					
Gender***					< 0.001
Men	29.61 %	29.00 %	43.70 %	38.97 %	
Women	70.39 %	71.00 %	56.30 %	61.03 %	
Age (years)***	56.98 (15.13)	52.22 (14.96)	48.11 (13.63)	48.89 (15.75)	< 0.001
Household size***	1.81 (1.02)	1.84 (1.03)	1.71 (0.99)	1.93 (1.10)	< 0.001
Household income ***	40,421 (27 179)	40,341 (27 091)	33,277 (26 453)	43,641 (28 266)	< 0.001
< $40,000	58.03 %	58.01 %	67.65 %	51.30 %	
$40,000 +	41.97 %	41.99 %	32.35 %	48.70 %	
Marital status***					< 0.001
Married/common-law	40.84 %	40.16 %	28.99 %	39.71 %	
Widowed, separated, divorced	34.26 %	29.40 %	30.67 %	26.25 %	
Single, never married	24.90 %	30.45 %	40.34 %	34.04 %	
Child < 12 yo					0.1623
No child	88.68 %	87.53 %	88.24 %	85.87 %	
At least one child	11.32 %	12.47 %	11.76 %	14.13 %	
Education					0.4524
High school or less	26.10 %	25.59 %	25.32 %	24.69 %	
High school diploma with post-sec.	14.38 %	14.38 %	10.97 %	13.56 %	
Partial post-sec. studies	7.59 %	9.37 %	8.02 %	7.27 %	
Post-sec. diploma or university	51.93 %	50.66 %	55.70 %	54.49 %	
Birthplace					0.0583
Canada & America	94.13 %	97.11 %	93.91 %	93.68 %	
Rest of the world	5.87 %	2.89 %	6.09 %	6.32 %	
**Self-assessment of health**					
Overall health***					< 0.001
Very good to excellent	22.89 %	23.88 %	19.33 %	28.42 %	
Good	32.76 %	34.65 %	31.93 %	38.43 %	
Fair to bad	44.40 %	41.47 %	48.74 %	33.16 %	
Mental health***					< 0.001
Very good to excellent	24.97 %	24.32 %	16.74 %	25.86 %	
Good	43.13 %	43.07 %	39.48 %	40.75 %	
Fair to bad	31.90 %	32.61 %	43.78 %	33.39 %	
Perceived stress in life					0.2403
Not very to not at all stressful	25.00 %	23.24 %	19.75 %	22.57 %	
A little stressful	33.99 %	31.87 %	38.66 %	35.79 %	
Fairly to extremely stressful	41.01 %	44.89 %	41.60 %	41.64 %	
Stress at work					0.1074
Not very to not at all stressful	16.38 %	16.43 %	21.15 %	17.55 %	
A little stressful	27.99 %	31.20 %	32.69 %	34.43 %	
Fairly to extremely stressful	55.63 %	52.37 %	46.15 %	48.02 %	
Health utility index*** (HUI3)	0.6221 (0.3255)	0.6593 (0.3145)	0.6365 (0.3037)	0.7116 (0.2813)	< 0.001
AIC: 971,747	BIC: 971,996				
Between group difference is significant at p: *<0.05, **<0.01; ***<0.001					

**Fig. 1:**
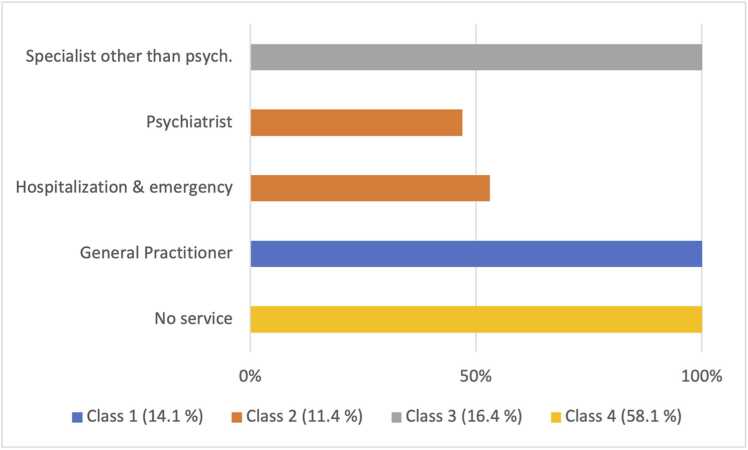
Distribution of respondents by services used.

The post-estimation distribution (Fig. 1) shows an almost exclusive distribution of classes among medical services, except for Class 2, which includes a combination of hospital and ED services and the consultation of a psychiatrist.

### 3.3 Latent profile analysis

**Fig. 2:**
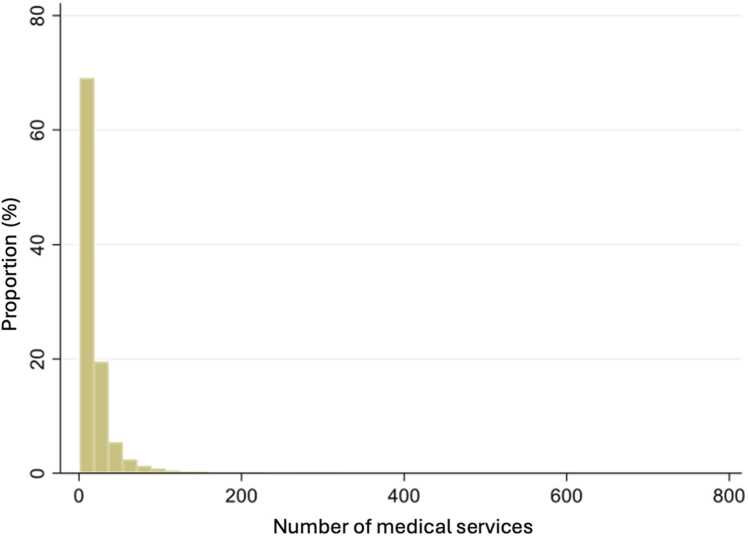
Distribution of the number of medical services.

**Fig. 3:**
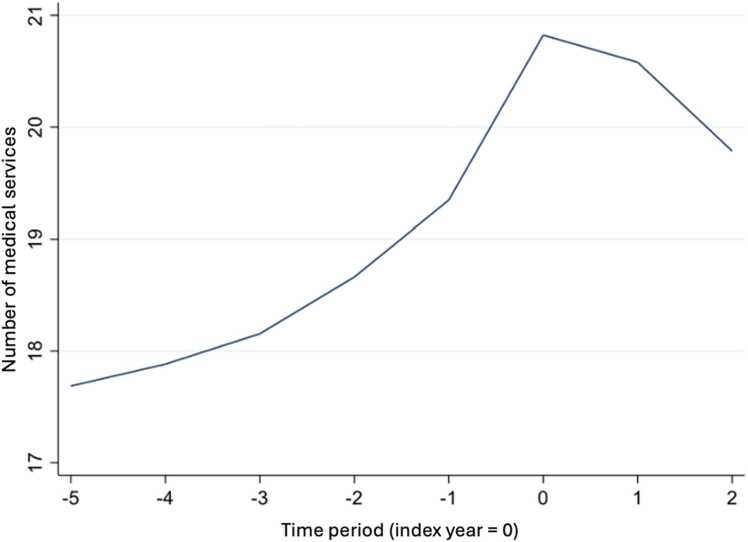
Annual trend of the number of medical services.

The distribution of medical services in Fig. 2 shows that close to 70 % of people did not receive a medical service during the study period. For those who did receive a medical service, we note in Fig. 3 that the number of services increased over time, going from 17 to 20 services with a peak in the year that the CCHS was completed.

Based on the AIC and BIC statistics, we selected the four-profile model for the medical services and the two-profile model for hospitalizations.

**Fig. 4:**
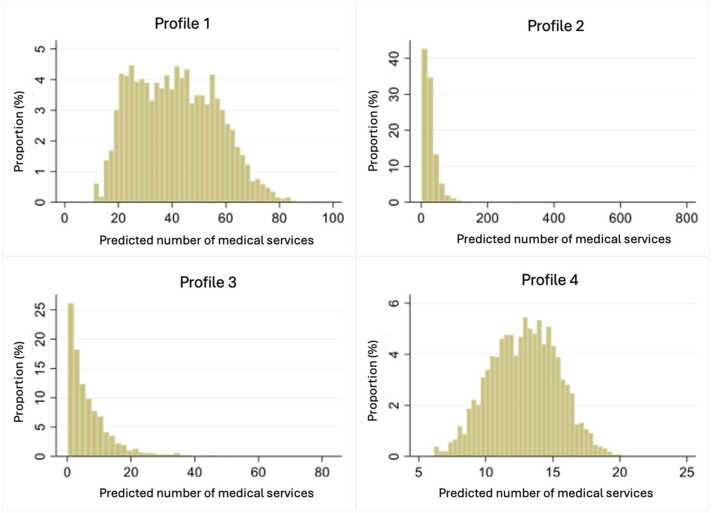
Distribution of the number of medical services per profile.

**Fig. 5:**
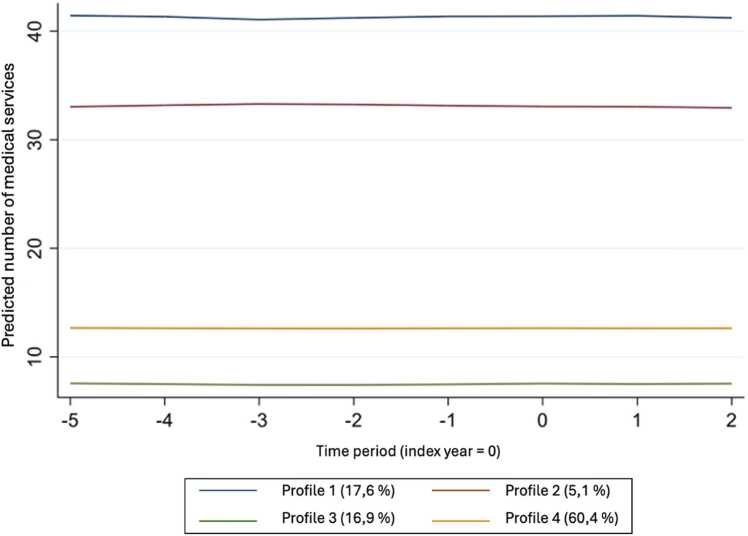
Annual trends of the number of medical consultations for each profile.

Profile 1 is characterized by a large consumption of medical services with an annual average of 41 (se:16), which represents over three visits per month, and 17.6 % of the study population. Profile 2 has an average annual consumption of 33 medical services per year and represents 5.1 % of the population. Profile 3 includes 16.9 % of the population and has an annual average of seven medical services used per year. The most populous profile was profile 4 with over 60 % of the study population and consisted of people with one medical service used. The distribution of the consumption of medical services across profiles is shown in Fig. 4. The averages are presented in Fig. 5.

The results of the regression in Table 5 show that women were more likely to have medical services in profiles 3 and 4, and less likely in profiles 1 and 2 (although not significant for profile 1). Age was a significant factor in having medical services regardless the profile, but it was not significant for profile 2. Respondents with higher revenue in profiles 1 and 4 were less likely to use medical services than those in profile 3. Compared to married or common-law relationships, widow/separated/divorced respondents in profile 3 and single/never married respondents in profile 4 were less likely to use medical services. However, those same respondents were more likely to use medical services in profiles 4 and 2, respectively. Only in profile 1, respondents who have at least one child under 12 were less likely to use medical services compared to those that did not. Overall, the education level had a mitigated impact on the use of medical services and did not exhibit significant results. In average, each respondent in profile 1 used medical services 41 times. This number totalled 33, 7 and 12 for profile 2, 3 and 4, respectively.

**Table 5:** Results of the regression on the profile for the number of medical services used.

	Profile 1 (17.6 %)		Profile 2 (5.1 %)		Profile 3 (16.9 %)		Profile 4 (60.4 %)	
	Coeff. (s.e.)	*P value*	Coeff. (s.e.)	*p*	Coeff. (s.e.)	*p*	Coeff. (s.e.)	*p*
Gender (ref=men)								
Women	−0.02 (0.04)	0.629	−0.56 (0.13)	0.000	1.39 (0.14)	0.000	0.16 (0.02)	0.000
Age	0.01 (0.00)	0.000	0.01 (0.00)	0.125	0.06 (0.00)	0.000	0.01 (0.00)	0.000
Household income	−0.21 (0.02)	0.000	0.04 (0.05)	0.352	0.08 (0.04)	0.032	−0.07 (0.01)	0.000
Marital status (ref = common law/ married)								
Widow/separated/ divorced	0.09 (0.05)	0.065	0.09 (0.21)	0.683	−0.32 (0.13)	0.013	0.03 (0.03)	0.368
Single/ never married	0.02 (0.05)	0.715	0.49 (0.12)	0.000	−0.26 (0.14)	0.063	−0.05 (0.02)	0.037
Child < 12 (ref = None)								
At least one	−0.30 (0.08)	0.000	0.76 (0.22)	0.001	0.45 (0.17)	0.007	0.12 (0.03)	0.000
Highest education level attained (ref=high school not completed)								
High school diploma	0.05 (0.05)	0.376	−0.32 (0.16)	0.042	−0.03 (0.13)	0.849	−0.05 (0.03)	0.072
Partial post-sec.	−0.52 (0.13)	0.000	1.09 (0.23)	0.000	0.36 (0.17)	0.035	−0.03 (0.04)	0.381
Post-sec diploma or university	−0.07 (0.04)	0.109	−0.20 (0.13)	0.120	0.13 (0.11)	0.228	0.04 (0.02)	0.111
Household size	0.11 (0.03)	0.000	0.00 (0.07)	0.965	−0.09 (0.07)	0.183	0.01 (0.01)	0.545
Predicted value								
Average	41.29 (15.98)		33.11 (24.62)		7.48 (8.90)		12.63 (2.55)	
Probability	17.6 %		5.1 %		16.9 %		60.4 %	

n = 35,368; AIC = 267,487.5; BIC = 26,7919.3

**Fig. 6:**
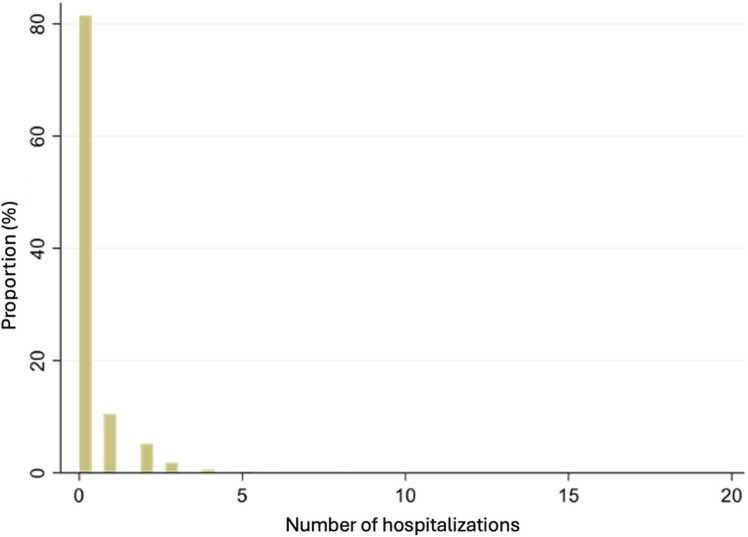
Distribution of the annual number of hospitalizations.

When examining hospitalizations, we note that about 20 % of the population had at least one hospitalization during the study period and 80 % had none (Fig. 6). The number of hospitalizations is under one per year per person.

For hospitalizations, we selected the model with two profiles. The results from the regression are reported in Table 6. The results show that, compared to men, women in profile 1 were less likely to be hospitalized, while those in profile 2 were more likely. Overall, respondents in profile 1 were 15 times more likely to be hospitalized than those in profile 2, though they represented 29 % of the total.

**Table 6:** Regression results on the number of hospitalizations.

		Profile 1 (28.8 %)		Profile 2 (71.2 %)	
		Coeff. (s.e.)	*P value*	Coeff. (s.e.)	*P value*
Gender	(ref = Men)				
	Women	−0.14 (0.03)	0.000	0.67 (0.08)	0.000
Age		0.01 (0.00)	0.000	−0.02 (0.00)	0.000
Household income		−0.05 (0.01)	0.000	−0.01 (0.03)	0.675
Marital status	(ref = Married/common-law)				
	Widow, separated, divorced	0.009 (0.03)	0.009	0.04 (0.09)	0.649
	Single, never married	−0.05 (0.04)	0.229	−0.08 (0.90)	0.320
Child < 12	(ref = None)				
	At least one	−33.64 (1e+04)	1.00	−18.60 (840)	0.982
Highest level of education	(ref = high school not completed)				
	High school	−0.018 (0.04)	0.000	−0.06 (0.11)	0.591
	Partial post-sec.	−0.18 (0.05)	0.000	−0.40 (0.14)	0.004
	Post-sec. diploma or university	−0.16 (0.03)	0.000	−0.16 (0.09)	0.063
Household size		0.06 (0.02)	0.005	0.07 (0.03)	0.013
Predicted value					
	Average	0.91 (0.003)		0.06 (0.001)	
	Probability	28.8 %		71.2 %	

n = 35,368; AIC = 47,797.8; BIC = 47,992.58

**Fig. 7:**
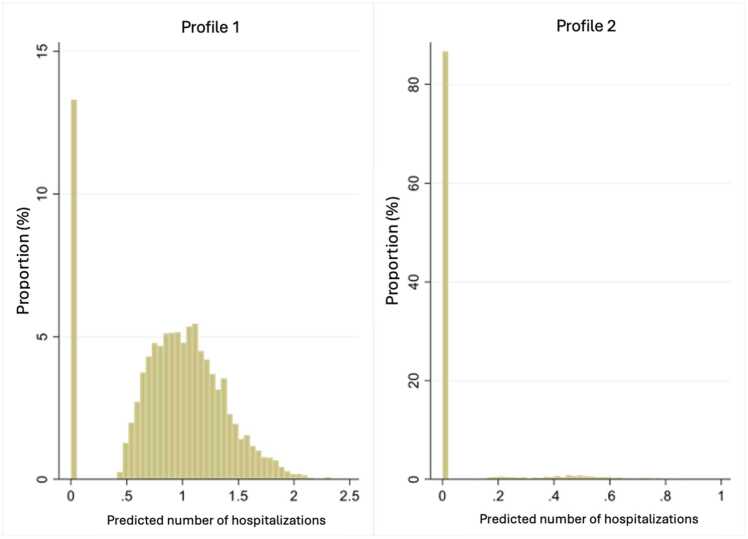
Distribution of the average annual number of hospitalizations for each profile.

**Fig. 8:**
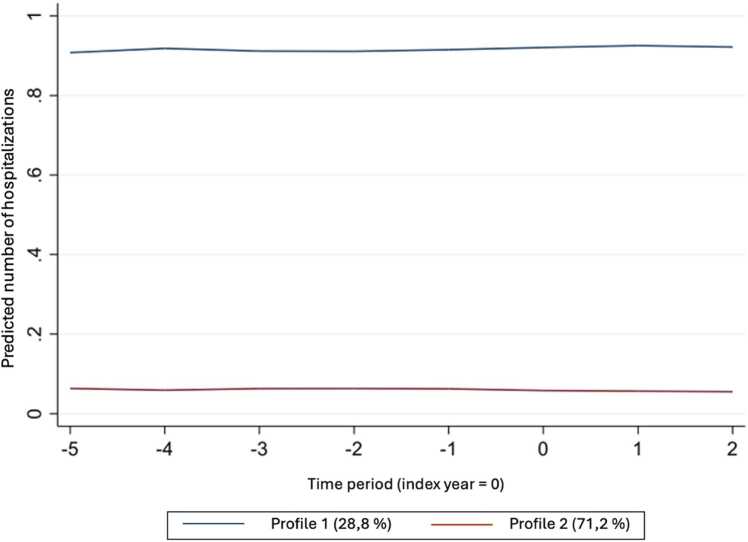
Annual trend in the number of hospitalizations for each profile.

The post-estimation distribution for each profile is shown in Fig. 7 and the trends in the number of hospitalizations can be seen in Fig. 8. In profile 1 about 14 % of people had no hospitalization, while in profile 2, over 80 % of people had no hospitalization.

## 4 Discussion

This study examined care trajectories using LCA and LPA methods for people who self-reported having been diagnosed with MD. Our results show the heterogeneity in the care trajectories with various utilization intensity levels, and utilization of different types of providers. The advantage of the LCA is that the classes can include different types of utilization. For instance, some people consult mainly with a general practitioner, while another class consults with a psychiatrist, and yet another with other specialists. The LCA showed a breakdown of the study population into four groups, while the LPA showed that overall, our sample was composed of four groups for medical service users and two groups for hospitalizations. One of the benefits of the LPA is that we can examine utilization intensity within a type of service. An important finding showed that in both LCA and LPA, the largest group (around 60 % of the population) consisted of people with null or very low utilization of services over the study period. This suggests that living with a mood disorder diagnosis does not necessarily translate into utilization of services, even though studies have found a higher utilization of services in this population compared to the general population [6], [7], [8]. There are various ways to interpret this large proportion of null utilization. First, the MD may be well controlled and not require frequent follow-up with a physician. The second hypothesis is that some people may be monitored by a provider whose services are not available in the administrative databases, whether covered by public insurance (social worker, mental health nurse, psychologist, peer support worker) or through a private insurance or out of pocket (psychologist). However, it is impossible to know how the use of such services would be distributed across classes. Third, the null or low utilization could reflect an underuse and barriers to care, which have been reported as being more prevalent in people with MD compared to the general population [37], [38]. They may also not be aware of services available or able to identify where or how to access services or obtain a referral [39]. While Quebec’s health care system provides full coverage (with co-payment or deductible) to a relatively wide range of services, there can still be barriers to services, such as transportation, arranging childcare, taking time off work or discomfort related to judgment, as MD are still stigmatized. We need to better promote the health services available to help people suffering from mood disorders to reduce the burden of the disease. The fact that ACL classes 1 and 2 have more women and older people implies that either these are the people most at risk of mood disorders, or that men and young adults are not using enough health services for their disorders. Thus, more specific research needs to be carried out in this area to better address the situation. It is important to consider that our study examined actual utilization of services and not health care needs. A study on the Canadian population using CCHS data reported that people with MD were more likely to report unmet health care needs [40]. We do not know the trajectories of the health needs of people and how much these are related to the utilization of services. This could be examined in future studies.

A study on Quebec adult patients’ care trajectories before the first schizophrenia diagnosis has highlighted five type of care trajectories [17]. Using state sequential analysis, the authors found that more than 77 % of patients “sought little health care,” including 17.5 % which “had no health care contact for mental disorders.” Another study [41] on individuals with anxiety disorder in Quebec categorized the patients into five care trajectories and found that more than half of them were low-care seeking. These results remain consistent with ours in that a large majority of patients are not in or have not sought contact with health care services, even if they are not the same mental illnesses.

### 4.1 Strength and limitations

This study has allowed to us to draw care trajectories for patients with mood disorders. We used two similar methods to highlight resource utilization and its intensity.

LCA and LPA are two methods that have the advantage of parametrically estimating latent groups, thus avoiding biases that could be related to researcher preferences. In addition, they allow several variables of interest to be considered simultaneously to determine the class of membership. The main drawback is that LCA and LPA only allow the estimation of average effects. In the case of no utilization of medical services, the presence of many 0 values could prevent optimal estimation of latent classes or profiles. On the other hand, these methods have the disadvantage of estimating many parameters that may be difficult to interpret.

This study highlights some methodological limitations worth noting. The first concerns the selection of individuals in our cohort, based on the respondent's self-report of having been diagnosed with MD. Some people may not want to report the presence of a mental illness because of the stigma they may experience, which could potentially reduce the real sample size of people suffering MD. However, missing some people does not mean that the results are biased. In addition, we think that our approach was justified as argued in another study [26], and the best alternative given the limitation above mentioned regarding diagnosis data. In fact, Pelletier et al. found that MD were underdiagnosed in Canada [42]. A comparative analysis of diagnosis-based and self-declared depression suggests that self-declaration was the most valid approach [43]. Self-reports were also considered valid for MD in a study on veterans in the United States [44].

The second limitation comes from the construction of the CCHS survey questionnaire. There was no way of differentiating between which kind of MD people were suffering. Since the question was stated as follow: “Do you have a mood disorder such as depression, bipolar disorder, mania, or dysthymia?”, it groups all types of MD. Though they were grouped under the common name of MD, these disorders do not have the same impact on a patient's daily life, and some are distinguished by their chronic nature (i.e., bipolar disorder), which may consequently influence their care trajectory. For instance, bipolar disorder, although less common than major depressive disorder, is considered more serious and persistent, which could be associated with higher services utilization [45]. However, there is overlapping symptomatology leading to frequent misdiagnosis between MD, and particularly between major depressive and bipolar disorders [45], [46], [47], [48], [49]. In fact, deep learning algorithms have recently been developed to distinguish these conditions [50]. Other tools have also recently been developed to distinguish bipolar and major depressive disorders [46], [47] or specifically for the diagnosis of bipolar disorder [49]. Hence, combining these conditions is aligned with the difficulty to accurately differentiate them.

Finally, another limitation is related to the medical-administrative data available for the analysis of the use of health care and services. Unfortunately, the data from the RAMQ databases are mainly of a medical nature, since they only include services covered by the RAMQ, and not services paid for by users or services covered by private insurance. It is expected that patients with MD use health care services not covered by the public health insurance plan. For instance, although some psychology services are covered by public insurance, most psychologists work in private practices and services are then covered by private insurance. In addition, some patients use support services provided by community-based organizations. Services that are paid for through private insurance or directly out of pocket by patients are not included in our study, nor are services delivered by community-based organizations, which are mostly funded by the government and whose services are generally free at the point of service. Other services covered by the public health insurance plan, but delivered by salaried health providers, are also not included in our databases. Having services provided by other providers would have enabled a more complete picture of the trajectories. However, their absence does not affect our findings regarding the medical and hospital services being used. Instead, having these additional data would support planning of health human resources, which in our study is limited to medical and hospital services.

## 5 Conclusion

With both methods, LCA and LPA, we identified a group of people with MD who have a null utilization of health services, and this group constituted over half of the study population in both cases. Although people with MD may require more health services than the general population, most of them are not high users. It is also possible that their needs are not well reflected in utilization of publicly insured covered services. Studying other services such as those delivered by psychologists could improve the modelling of care trajectories for people with MD.

## Funding

The project was funded by the Quebec Learning Health Systems Support Unit (Unité de Soutien SSA-Québec).

## Declaration of Competing Interest

The authors declare the following financial interests/personal relationships which may be considered as potential competing interests: Maude Laberge reports financial support was provided by Quebec Support Unit for Learning Health Systems. If there are other authors, they declare that they have no known competing financial interests or personal relationships that could have appeared to influence the work reported in this paper.
